# ZNF148 inhibits HBV replication by downregulating RXRα transcription

**DOI:** 10.1186/s12985-024-02291-4

**Published:** 2024-01-31

**Authors:** Xinyan Yao, Kexin Xu, Nana Tao, Shengtao Cheng, Huajian Chen, Dapeng Zhang, Minli Yang, Ming Tan, Haibo Yu, Peng Chen, Zongzhu Zhan, Siyi He, Ranran Li, Chunduo Wang, Daiqing Wu, Jihua Ren

**Affiliations:** 1 The Key Laboratory of Molecular Biology of Infectious Diseases designated by the Chinese Ministry of Education, Chongqing Medical University, Chong Yi Building, 1 YiXueYuan Road, Yuzhong District, Chongqing, 400016 China; 2 Department of Clinical Laboratory, Chongqing Traditional Chinese Medicine Hospital, Chongqing, China; 3 Chongqing Key Laboratory of Sichuan-Chongqing Co-construction for Diagnosis and Treatment of Infectious Diseases Integrated Traditional Chinese and Western Medicine, Chongqing Hospital of Traditional Chinese Medicine, Chongqing, China; 4 Department of Clinical Laboratory, Chongqing Emergency Medical Center, Chongqing University Central Hospital, Chongqing, China

**Keywords:** HBV, ZNF148, Transcription factor, RXRα, cccDNA

## Abstract

**Background:**

Progressive hepatitis B virus (HBV) infection can result in cirrhosis, hepatocellular cancer, and chronic hepatitis. While antiviral drugs that are now on the market are efficient in controlling HBV infection, finding a functional cure is still quite difficult. Identifying host factors involved in regulating the HBV life cycle will contribute to the development of new antiviral strategies. Zinc finger proteins have a significant function in HBV replication, according to earlier studies. Zinc finger protein 148 (ZNF148), a zinc finger transcription factor, regulates the expression of various genes by specifically binding to GC-rich sequences within promoter regions. The function of ZNF148 in HBV replication was investigated in this study.

**Methods:**

HepG2-Na^+^/taurocholate cotransporting polypeptide (HepG2-NTCP) cells and Huh7 cells were used to evaluate the function of ZNF148 in vitro. Northern blotting and real-time PCR were used to quantify the amount of viral RNA. Southern blotting and real-time PCR were used to quantify the amount of viral DNA. Viral protein levels were elevated, according to the Western blot results. Dual-luciferase reporter assays were used to examine the transcriptional activity of viral promoters. ZNF148’s impact on HBV in vivo was investigated using an established rcccDNA mouse model.

**Results:**

ZNF148 overexpression significantly decreased the levels of HBV RNAs and HBV core DNA in HBV-infected HepG2-NTCP cells and Huh7 cells expressing prcccDNA. Silencing ZNF148 exhibited the opposite effects in both cell lines. Furthermore, ZNF148 inhibited the activity of HBV ENII/Cp and the transcriptional activity of cccDNA. Mechanistic studies revealed that ZNF148 attenuated retinoid X receptor alpha (RXRα) expression by binding to the RXRα promoter sequence. RXRα binding site mutation or RXRα overexpression abolished the suppressive effect of ZNF148 on HBV replication. The inhibitory effect of ZNF148 was also observed in the rcccDNA mouse model.

**Conclusions:**

ZNF148 inhibited HBV replication by downregulating RXRα transcription. Our findings reveal that ZNF148 may be a new target for anti-HBV strategies.

**Supplementary Information:**

The online version contains supplementary material available at 10.1186/s12985-024-02291-4.

## Introduction

Hepatitis B virus (HBV) infection is a serious worldwide public health concern that has the potential to cause serious liver conditions such as primary hepatocellular carcinoma and liver cirrhosis (HCC) [1]. Hepatitis B vaccine has resulted in a continual decline in the number of people with HBV infection; nonetheless, there are still over 257 million HBV-positive people in the globe, and it is predicted that by 2030, the number of people who die from HBV-related illnesses will rise to 1,149,000 [2]. The currently available therapeutic drugs, including nucleotide analogs and interferon, can efficiently inhibit viral replication but cannot achieve a functional cure for chronic hepatitis B (CHB) [3]. Thus, the necessity for developing new, effective treatments for HBV is increasing.

Partially double-stranded relaxed circular DNA (rcDNA) makes up the 3.2 kb genome of HBV, a member of the Hepadnaviridae family. In host hepatocytes, HBV utilizes the Na^+^/taurocholate cotransporting polypeptide (NTCP) receptor for entry and then releases its rcDNA genome, which can subsequently be converted into covalently closed circular DNA (cccDNA) in the nucleus. cccDNA represents a genetically stable configuration of the viral genome and serves as a template for both replication and transcription. The viral genome has four promoters (the core promoter [Cp], Sp1, Sp2, and Xp) and two transcriptional enhancer elements (I and II) from which 4 major viral RNAs, namely, the 3.5, 2.4, 2.1 and 0.7 kb mRNAs, are transcribed [4]. According to recent studies, host factors including hepatocyte nuclear factor 4α (HNF4α) [5], the tumor suppressor protein p53 [6], zinc fingers and homeoboxes 2 (ZHX2) [7], CCAAT/enhancer-binding protein (C/EBP) [8] and signal transducer and activator of transcription family member 1 (STAT1), significantly regulate the transcription and replication of HBV through interactions with HBV promoters and enhancers [9]. Consequently, modifying the way that host factors and HBV interact may represent a novel treatment strategy for controlling HBV infection.

Zinc finger proteins (ZFPs) are transcription factors that are essential for controlling the expression of certain genes. They have DNA-binding domains that resemble fingers. These proteins exhibit diverse zinc finger structures, including Cys2His2 (C2H2)-like, Zn2/Cys6, TAZ2 domain-like, Treble clef, zinc ribbons, zinc-binding loop, Gag knuckle, and metallothionein motifs [10]. These distinct zinc finger motifs confer a range of biological functions on ZFPs, such as functions in cell growth and death, metabolism, and autophagy [11]. It has recently been discovered that a number of ZFPs regulate the transcription and replication of HBV. Zinc-finger E-box binding homeobox 2 (ZEB2) has been discovered to bind to HBV promoter regions and thereby limit the transcriptional activity of HBV cccDNA [12]. Krüppel-like factor (KLF15) promotes HBV transcription and replication by binding to the HBV S and C promoters [13]. Moreover, earlier research has demonstrated that the HBV genome contains ZFP binding sites [14], supplying a structural foundation for ZFPs’ function in adjusting the HBV life cycle. Among ZFPs, zinc finger protein 148 (ZNF148), a member of the Krüppel-like (C2H2) ZFP family, plays an important role in regulating gene expression by binding to GC-rich sequences in the promoter regions of genes such as PTX3 [15], CTNNB1 [16], Bak [17], ID1/3 [18] and pdcd4 [19], which are involved in the development of glioma, colorectal cancer, HCC and breast cancer. Nevertheless, not much research has been done to find out if ZNF148 plays a role in the HBV life cycle.

This study clarified the significance of ZNF148 in HBV transcription and replication. ZNF148 overexpression led to significant decreases in HBV RNA and HBV core DNA levels. Further mechanistic investigations revealed that ZNF148 hinders the activity of HBV ENII/Cp by suppressing the expression of RXRα, consequently impeding the transcriptional activity of cccDNA. In addition, in the relaxed covalently closed circular DNA (rcccDNA) mouse model, ectopic expression of ZNF148 reduced the levels of HBV markers. These results demonstrate ZNF148 as a potential therapeutic target for the treatment of HBV infection and provide fresh insights into the function of host components in the HBV life cycle.

## Materials and methods

### Cell culture and antibodies

HepAD38 and HepG2-NTCP cells were gifts from Xiamen University. HepAD38 cells were cultured in Dulbecco’s modified Eagle’s medium (DMEM) supplemented with 10% fetal bovine serum (FBS) and 400 mg/ml G418 (345,810, Merck Millipore, Germany). HepG2-NTCP cells were maintained in DMEM supplemented with 10% FBS and 2 μg/ml doxycycline (Dox; Solarbio, China). Huh7 cells were obtained from the Cell Bank of the Chinese Academy of Sciences (Shanghai, China). Primary human hepatocytes (PHHs) were purchased from ScienCell Research Laboratories. Huh7 cells were cultured in DMEM supplemented with 10% FBS, and PHHs were maintained in a hepatocyte medium. All cells mentioned above were cultured in an atmosphere at 37 °C and 5% CO_2_. The mouse anti-ZNF148 (sc137171) antibody was purchased from Santa Cruz Biotechnology (United States). The rabbit anti-RXRα (21218-1-AP) antibody was obtained from Proteintech (United States). The mouse anti-HBc antibody was a kind gift from Dr. Xuefei Cai (Chongqing Medical University, China). The mouse anti-GAPDH (MB001) antibody was obtained from Bioworld (United States).

### Animal experiments

C57BL/6 mice were obtained from the Laboratory Animal Center of Chongqing Medical University (China), and 6- to 8-week-old male mice were used in the experiments. Four micrograms of precursor cccDNA (precccDNA) and the Cre plasmid were diluted with PBS and delivered into the mice by hydrodynamic injection. After one week, all the mice were randomly assigned to one of two groups: the negative control group (AAV-EGFP, 2 × 10^11^, *n* = 8) and the treatment group (AAV-ZNF148, 2 × 10^11^, *n* = 8). Blood samples were collected from the mice every 4 d, and serum was obtained after centrifugation at 2000 × g for 15 min for further study. After twenty d, the mice were sacrificed, and liver tissues and blood were collected for further research. The protocol was approved by the Laboratory Animal Center of Chongqing Medical University.

### Virus collection and infection

HBV stocks were produced from the culture supernatant of HepAD38 cells. In brief, the HBV stocks were precipitated from the medium by mixing with 5% PEG8000 overnight at 4 °C. The mixture was subsequently centrifuged at 4,000 rpm for 30 min. The particles were resuspended in Opti-MEM. The titer of the HBV particles was measured by real-time PCR. For infection, cells preseeded in wells were cultured in a normal medium for 24 h. Then, the cells were cultured in PMM supplemented with 2 μg/ml Dox for 24 h and subsequently infected with 1000 HBV genomes per cell (multiplicity of infection, MOI = 1000) diluted in William’s medium in the presence of 4% PEG8000. After 24 h, the cells were washed three times and cultured in a maintenance medium supplemented with 2.5% DMSO.

### Quantitative reverse transcription-PCR (qRT-PCR)

Total cellular RNA was extracted using TRNzol reagent (TIANGEN, China), and cDNA synthesis was performed using 1 μg of total RNA with the FastKing RT Kit (TIANGEN, China). qPCR was conducted to analyze the target RNA using Fast Start Universal SYBR Green Master (Bio-Rad, United States). The relative levels of viral RNA were determined using the 2^−ΔΔCT^ method, with β-actin mRNA serving as the internal control. The primers utilized in this study are listed in Supplementary Table 1.

### ELISA

The concentrations of secreted HBeAg and HBsAg in the cell supernatant were measured using a commercial diagnostic ELISA kit (Kehua Bio-engineering, Shanghai). The measured samples included both negative and positive controls for accurate comparison and analysis of the samples.

### HBV core DNA extraction and real-time PCR

Cells were washed with PBS and were then lysed in 500 μl of lysis buffer at 37 °C for 15 min. After centrifugation, the nuclei were removed, and the supernatant was incubated with DNase I and MgCl_2_ at 37 °C for 4 h to digest free DNA. HBV core capsids were precipitated using 5% PEG8000, and the precipitate was incubated with proteinase K overnight at 45 °C to release the HBV core DNA. Then, the HBV core DNA was extracted with phenol/chloroform, precipitated with ethanol, and dissolved in ddH_2_O. Quantification of HBV core DNA was performed using real-time PCR.

### HBV cccDNA extraction and analysis

Equal numbers of cells were collected in Eppendorf (EP) tubes and lysed in 500 μl of SDS lysis buffer at 37 °C for 20 min. Then, 125 μl of 2.5 M KCl was added to each tube, the mixture was inverted ten times, and the solution was incubated at 4 °C overnight. The next d, after centrifugation at 14,000 × g for 30 min, the supernatant was collected. The DNA was purified using phenol/chloroform and precipitated with ethanol. For HBV cccDNA detection, the extracted DNA was heated at 80 °C for 5 min to denature rcDNA and double-stranded DNA (dsDNA) and incubated with exonuclease V (M0345S, New England Biolabs, MA, United States) at 37 °C for 30 min. PCR with TaqMan probes was used to measure the level of cccDNA with the specific primers listed in Supplementary Table 1.

### Southern blotting

The HBV replicative intermediates extracted from cells were separated on 0.9% agarose gels and denatured using an alkaline solution. Subsequently, the DNA was transferred onto a nylon membrane (Roche, Mannheim, Germany) via a capillary siphon and fixed through UV crosslinking. Following prehybridization, the membrane was subjected to hybridization with a digoxigenin (DIG)-labeled probe. Next, the membrane was blocked and incubated with an anti-DIG antibody. The resulting signals on the membrane were visualized by exposure to film.

### Western blotting

Total cellular protein was extracted using RIPA lysis buffer supplemented with protease inhibitors. The protein concentration was determined using a BCA protein assay kit. Subsequently, 30 μg of protein was denatured at 95 °C for 10 min and separated via SDS‒PAGE. The separated proteins were then transferred onto a polyvinylidene fluoride membrane. The membrane was blocked with 5% skim milk for 2 h and incubated overnight at 4 °C with the appropriate primary antibodies. After washing with TBS-T, the membrane was incubated with the corresponding secondary antibodies for 2 h. The signals of the protein bands were visualized using an ECL Western blot reagent (Millipore, MA, United States).

### Northern blotting

Total cellular RNA was analyzed using the DIG Northern Starter Kit following the manufacturer’s protocol. HBV RNA was separated on a 1.4% formaldehyde-agarose gel and transferred onto nylon membranes overnight. After fixation through UV crosslinking, a DIG-labeled HBV RNA probe was hybridized to the membranes at 68 °C overnight. In the following d, the membranes were washed sequentially with 2× and 0.1× SSC solutions containing 0.1% SDS. Subsequently, the membranes were incubated in a blocking buffer containing an anti-DIG antibody for 30 min at 37 °C. The RNA signal was visualized by exposing the membranes to X-ray film.

### Luciferase reporter assay

Cells were transfected with reporter plasmids and the other indicated plasmids, and pRL-TK was used as an internal control. Thirty-six h after transfection, the cells were lysed in lysis buffer, and luciferase activity was measured via a dual luciferase reporter assay kit (Promega, United States).

### Chromatin immunoprecipitation (ChIP)

The ChIP assay was conducted following the manufacturer’s protocol (Merck Millipore, Germany) with slight modifications. Cells were collected, suspended in 500 μl of cell lysis buffer, and incubated on ice for 15 min. After centrifugation, the nuclei were precipitated and fixed using 1% formaldehyde. The cross-linked nuclei were collected by centrifugation and further resuspended in a nuclear lysis buffer. The nuclear lysate was then sonicated to obtain fragments ranging from 200 to 1000 bp in length. After centrifugation, 50 μl of the supernatant was diluted to 500 μl with dilution buffer. The diluted supernatant was incubated with the specified antibodies overnight. Next d, the complexes were washed with a wash buffer and eluted with an elution buffer. The obtained immunoprecipitated chromatin was analyzed by real-time PCR. GAPDH was used as a control gene.

### Nascent RNA synthesis assay

Cells were incubated with a medium containing 0.2 mM 5-ethynyl uridine (EU). After 24 h, cellular RNA was extracted using an RNeasy Plus Mini Kit (QIAGEN, United States). The EU-labeled RNA was subjected to biotinylation via a click reaction. Streptavidin-coated beads were used to capture the biotinylated RNA. cDNA was subsequently synthesized with a FastKing RT Kit (TIANGEN, China). After incubation at 85 °C for 5 min, the cDNA on the beads was released and analyzed via real-time PCR using Fast Start Universal SYBR Green Master (Bio-Rad, United States).

### Immunohistochemistry

Liver tissues were embedded in paraffin and heated in sodium citrate buffer for 20 min after deparaffinization and rehydration. The sections were treated with endogenous hydrogen peroxide blocking solution (Beyotime, Shanghai, China) and blocked with ovine serum (ZSGB-Bio, Beijing, China) prior to incubation with antibodies specific for HBcAg or HBsAg at 4 °C overnight. The next d, the sections were incubated with a secondary antibody for one h. Diaminobenzidine (DAB) solution was used to detect tissue immunoreactivity (Dako, CA), and hematoxylin was used for counterstaining.

### Statistical analysis

Data from at least three independent experiments are presented as the means ± SDs. Statistical analysis was performed using the Student’s unpaired *t*-test or the nonparametric Mann–Whitney U test. All the statistical analyses were carried out using GraphPad Prism 8.0 software. A value of *P* < 0.05 was considered to indicate statistical significance (*P < 0.05, **P < 0.01).

## Results

### ZNF148 overexpression inhibits hepatitis B virus transcription and replication

**Fig. 1:**
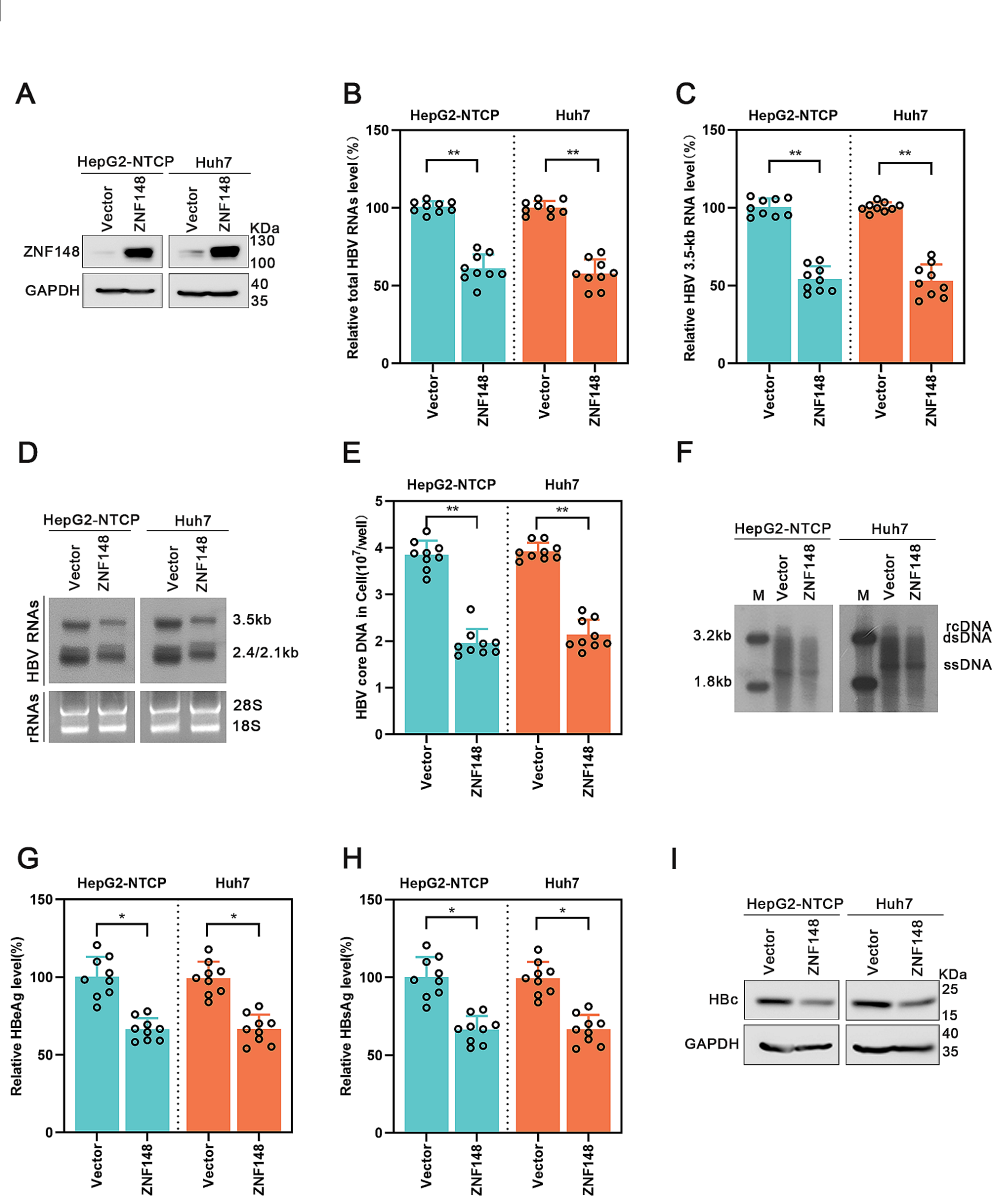
Overexpression of ZNF148 represses HBV transcription and replication. The vector or the ZNF148 plasmid was transfected into HepG2-NTCP cells infected with HBV and into Huh7 cells transfected with prcccDNA/Cre plasmids. The cells were harvested after 5 d. (**A**) The efficiency of ZNF148 overexpression was confirmed by Western blot analysis. (**B**-**D**) Real-time PCR and Northern blot analyses revealed significant reductions in the levels of total HBV RNA and 3.5-kb HBV RNA in cells overexpressing ZNF148. (**E**-**F**) Decreased levels of HBV core DNA in ZNF148-overexpressing cells were shown by real-time PCR and Southern blotting. (**G**-**H**) ZNF148 overexpression reduced the concentrations of HBsAg and HBeAg, as measured by ELISA. (**I**) The results of Western blot analysis confirmed the decreased level of the HBc protein in ZNF148-overexpressing cells. *P < 0.05, **P < 0.01

Zinc finger proteins are key regulators of the viral life cycle and important targets for drug development [20]. ZNF148 is a transcription factor that regulates gene expression by binding to GC-rich gene promoters [21]. The HBV genome also contains a ZFP binding domain [14]. Interestingly, in HBV-infected cells and mice, the expression of ZNF148 is down-regulated (Supplementary Figure S1), which suggests that ZNF148 may be related to HBV replication. To investigate the potential role of ZNF148 in the HBV life cycle, we transfected a plasmid expressing ZNF148 into HBV-infected HepG2-NTCP cells and Huh7 cells expressing prcccDNA. The results of Western blot analysis confirmed the efficiency of ZNF148 overexpression (Fig. 1A). Subsequently, we observed that overexpression of ZNF148 resulted in a decrease in the levels of HBV RNAs (Fig. 1B, C). Northern blot analysis further demonstrated the role of ZNF148 in decreasing 3.5, 2.4, and 2.1-kb HBV RNAs (Fig. 1D). Moreover, high expression of ZNF148 reduced the HBV core DNA level, as shown by real-time PCR and Southern blot analyses (Fig. 1E, F). Consistent with these findings, the concentrations of secreted HBeAg and HBsAg in the supernatant, and HBc protein levels were reduced by ZNF148 overexpression in both cell lines (Fig. 1G, H, I). Overall, these findings suggest that ZNF148 markedly represses HBV life cycle.

### ZNF148 silencing facilitates HBV transcription and replication

**Fig. 2:**
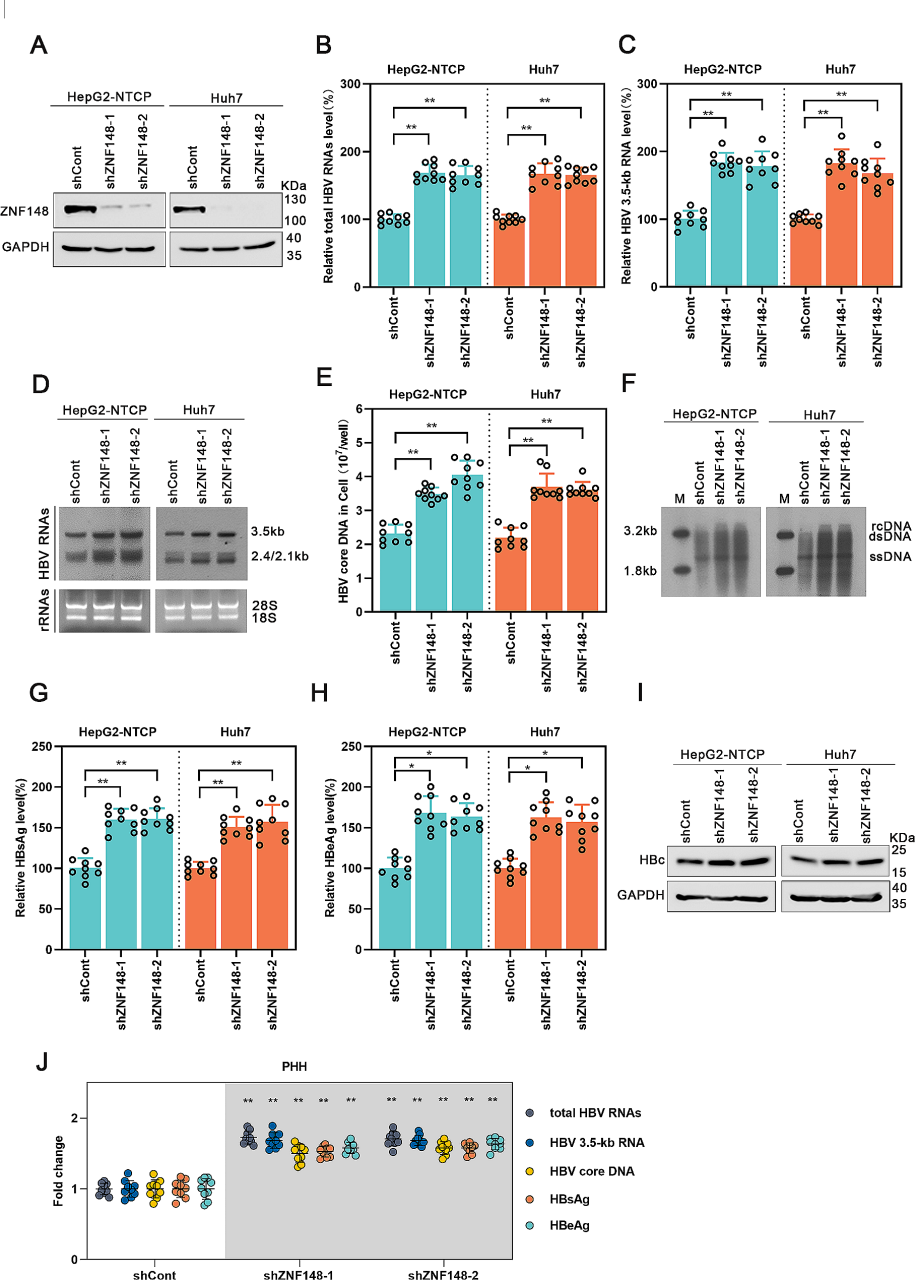
Silencing of ZNF148 promotes HBV transcription and replication. Lentiviruses expressing shRNA targeting ZNF148 or the control shRNA (shCont) were transduced into HepG2-NTCP cells infected with HBV or Huh7 cells transfected with prcccDNA/Cre plasmids. The cells were harvested after 5 d. (**A**) Western blot analysis was used to determine the efficiency of ZNF148 silencing. (**B**-**D**) ZNF148 silencing increased HBV RNA levels, as demonstrated by real-time PCR and Northern blotting. (**E**-**F**) Knockdown of ZNF148 increased the level of HBV core DNA, as determined by real-time PCR and Southern blotting. (**G**-**H**) Suppression of ZNF148 resulted in increases in the HBsAg and HBeAg concentrations, as measured by ELISA. (**I**) Western blot analysis was performed to measure the level of the HBc protein. (**J**) The abundances of HBV RNAs, DNA, HBsAg, and HBeAg in PHHs were quantified by real-time PCR and ELISA. *P < 0.05, **P < 0.01

To further examine the functional role of endogenous ZNF148 in HBV transcription and replication, we employed shZNF148-1 and shZNF148-2 to silence ZNF148 in HepG2-NTCP and Huh7 cells. The results of Western blot analysis confirmed that the expression of ZNF148 was significantly reduced by shZNF148-1 and shZNF148-2 (Fig. 2A). Real-time PCR and Northern blot analyses revealed notable increases in HBV RNA levels upon ZNF148 silencing (Fig. 2B, C, D). Furthermore, ZNF148 silencing resulted in a significant increase in the HBV core DNA level, as shown by real-time PCR and Southern blot analyses (Fig. 2E, F). The concentrations of secreted HBeAg and HBsAg in the supernatant were elevated upon ZNF148 silencing in both cell lines (Fig. 2G, H). The results of Western blot analysis showed that silencing ZNF148 led to a decrease in the HBc protein level (Fig. 2I). Additionally, we performed ZNF148 knockdown in PHHs; consistent with the above results, the HBV RNA, core DNA, HBsAg, and HBeAg levels were significantly increased (Fig. 2J). These results suggest that ZNF148 silencing promotes HBV transcription and replication.

### ZNF148 inhibits the transcriptional activity of HBV cccDNA

**Fig. 3:**
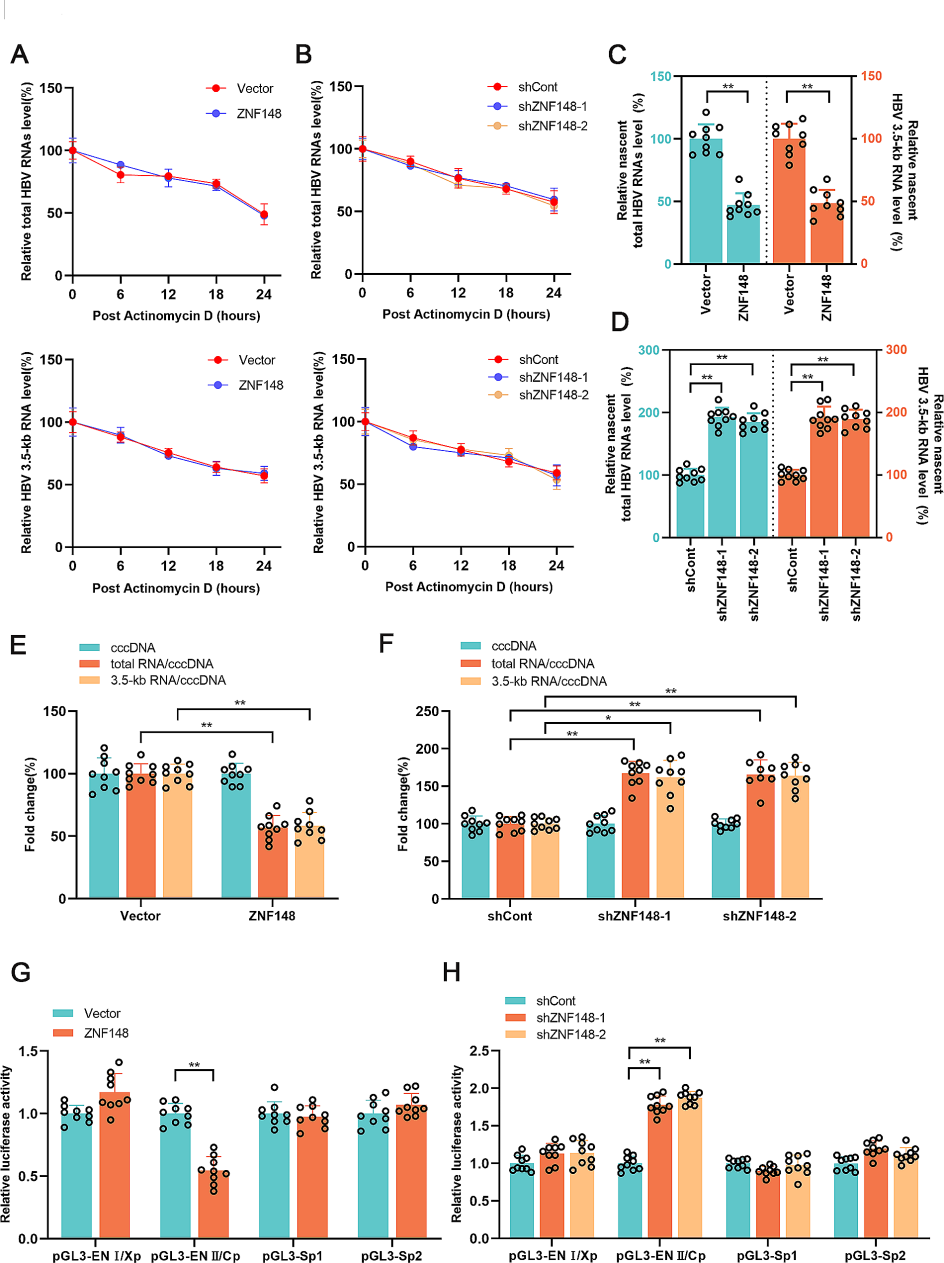
ZNF148 inhibits the transcriptional activity of HBV cccDNA. (**A**-**B**) HepG2-NTCP cells were infected with HBV and were then transfected with the vector/ZNF148 plasmid or infected with lentivirus expressing shCont/shZNF148. After 5 **d**, the cells were treated with actinomycin D (5 μg/ml) and harvested at the indicated times. (**C**-**D**) The cells were treated with 0.2 mM EU and harvested after 24 h. The amount of newly synthesized EU-labeled RNA was quantified via real-time PCR. (**E**-**F**) The ratios of total RNA and 3.5-kb RNA to cccDNA are considered markers of HBV cccDNA transcriptional activity. The level of HBV cccDNA was measured by real-time PCR. (**G**-**H**) Luciferase reporter plasmids containing the HBV promoter sequence were cotransfected with the vector/ZNF148 plasmid or transfected in combination with infection with lentivirus expressing shCont or shZNF148 into Huh7 cells. After 36 h, luciferase activity was measured via **a** dual luciferase assay. *P < 0.05, **P < 0.01

It was determined that ZNF148 acts as an inhibitory factor in HBV transcription. To investigate the underlying mechanism, we treated HepG2-NTCP cells with actinomycin D to block RNA synthesis. Cellular products were collected at 6-h intervals to measure HBV RNA levels. ZNF148 did not affect the half-life of HBV total HBV RNA or 3.5-kb HBV RNA (Fig. 3A, B), suggesting that ZNF148 does not affect HBV RNA stability. In addition, the results of the nascent RNA synthesis assay indicated that the levels of newly synthesized total HBV RNA and 3.5-kb HBV RNA were elevated upon ZNF148 overexpression and reduced upon ZNF148 knockdown (Fig. 3C, D). These findings suggest that ZNF148 affects the synthesis but not the stability of HBV RNA. We further investigated whether ZNF148 regulates HBV cccDNA synthesis or transcription. Neither overexpression nor silencing of ZNF148 affected the level of HBV cccDNA (Fig. 3E, F). The ratios of total RNA and 3.5-kb RNA to cccDNA are considered markers of HBV cccDNA transcriptional activity. We found that ZNF148 overexpression significantly reduced the transcriptional activity of HBV cccDNA (Fig. 3E). Consistent with this finding, ZNF148 silencing increased the transcriptional activity of HBV cccDNA (Fig. 3F). ZNF148 is a well-known transcription factor. We thus speculated that ZNF148 might regulate the transcriptional activity of HBV cccDNA. The dual luciferase reporter assay revealed that ZNF148 overexpression, but not ZNF148 silencing, suppressed the activity of HBV ENII/Cp (Fig. 3G, H). However, ZNF148 overexpression or silencing had only minor effects on the activity of HBV ENI/Xp, Sp1, and Sp2 (Fig. 3G, H). These findings suggest that ZNF148 may regulate HBV cccDNA transcription by inhibiting the activity of HBV ENII/Cp.

### ZNF148 suppresses RXRα expression

**Fig. 4:**
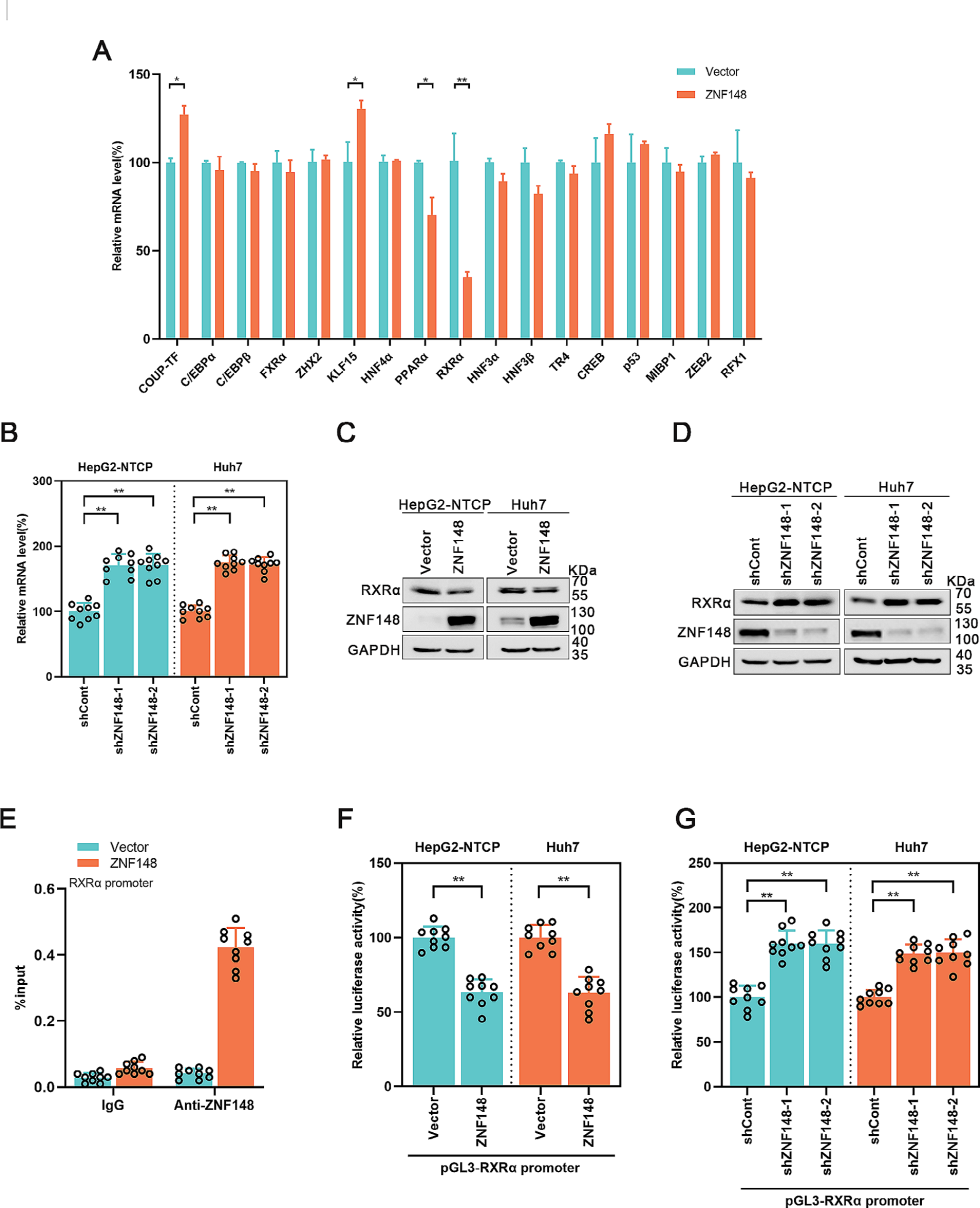
ZNF148 suppresses the expression of RXRα. (**A**) The mRNA levels of various relevant transcription factors in ZNF148-overexpressing Huh7 cells were examined by real-time PCR. (**B**) The mRNA level of RXRα in HepG2-NTCP and Huh7 cells infected with lentivirus expressing shCont/shZNF148 was measured by real-time PCR. (**C**-**D**) The protein level of RXRα in HepG2-NTCP and Huh7 cells transfected with the vector/ZNF148 plasmid or infected with lentivirus expressing shCont/shZNF148 were determined by Western blotting. (**E**) Huh7 cells were transfected with the vector/Flag-ZNF148 plasmid. After 72 h, the cells were harvested and subjected to immunoprecipitation with IgG or an anti-Flag antibody. The level of the RXRα promoter was measured using real-time PCR. (**F**-**G**) A dual luciferase reporter assay was performed to test the activity of the RXRα promoter. *P < 0.05, **P < 0.01

Considering that ZNF148 is a zinc finger transcription factor, we proposed that ZNF148 might regulate HBV transcription by binding to its promoter region directly. We generated a ZNF148 mutant (ZNF148^mut^) by deleting the zinc finger motif of ZNF148. ZNF148^mut^ had little effect on the levels of HBV RNAs, HBV DNA, the secreted HBeAg and HBsAg in the supernatant and the HBc protein (Supplementary Figure S2), these findings suggest that the function of ZNF148 inhibiting HBV replication may depends on its zinc finger structure. Moreover, we utilized the JASPAR database to predict and identify a putative ZNF148 binding site within the HBV ENII/Cp region. Then, we generated an ENII/Cp mutant (HBV ENII/Cp Mut, in which the ZNF148 binding site was mutated) in pGL3, but luciferase reporter assays showed that ZNF148 had the same effect on the activity of HBV ENII/Cp Mut as it did on the activity of HBV ENII/Cp wild-type (WT) (Supplementary Figure S3). Furthermore, the ChIP assay showed that ZNF148 exhibited minimal binding to the HBV genome (Supplementary Figure S4). These data suggested that the regulatory effect of ZNF148 on HBV may not be direct. We subsequently examined a series of transcription factors that are involved in HBV transcription, such as cAMP response element-binding protein (CREB), farnesoid X receptor alpha (FXRα), ZHX2, p53, C/EBP, and retinoid X receptor alpha (RXRα). Real-time PCR showed that ZNF148 overexpression affected the mRNA levels of COUP-TF, KLF15, PPARα, and RXRα, among which the RXRα mRNA level exhibited the greatest decrease in ZNF148-overexpressing cells (Fig. 4A). Moreover, ZNF148 silencing increased the RXRα mRNA level (Fig. 4B). The protein level of RXRα was also decreased in ZNF148-overexpressing cells and was increased in ZNF148-silenced cells (Fig. 4C, D). In addition, the promoter region of RXRα contains a binding site for ZNF148, according to the JASPAR database. The ChIP assay results confirmed that ZNF148 binds to the promoter region of RXRα (Fig. 4E). Therefore, we further investigated whether RXRα is involved in the regulation of HBV transcription by ZNF148. Via a dual luciferase assay, we observed that overexpression of ZNF148 suppressed the activity of the RXRα promoter. Conversely, silencing ZNF148 increased the activity of the RXRα promoter (Fig. 4F, G). Additionally, we identified the ZNF148 binding site in the RXRα promoter with software and mutated this binding site, which abolished the effect of ZNF148 on the activity of the RXRα promoter (Supplementary Figure S5). These results suggest that ZNF148 decreases RXRα expression by inhibiting the activity of its promoter.

### ZNF148 inhibits HBV replication by suppressing the binding of RXRα to HBV ENII/Cp

**Fig. 5:**
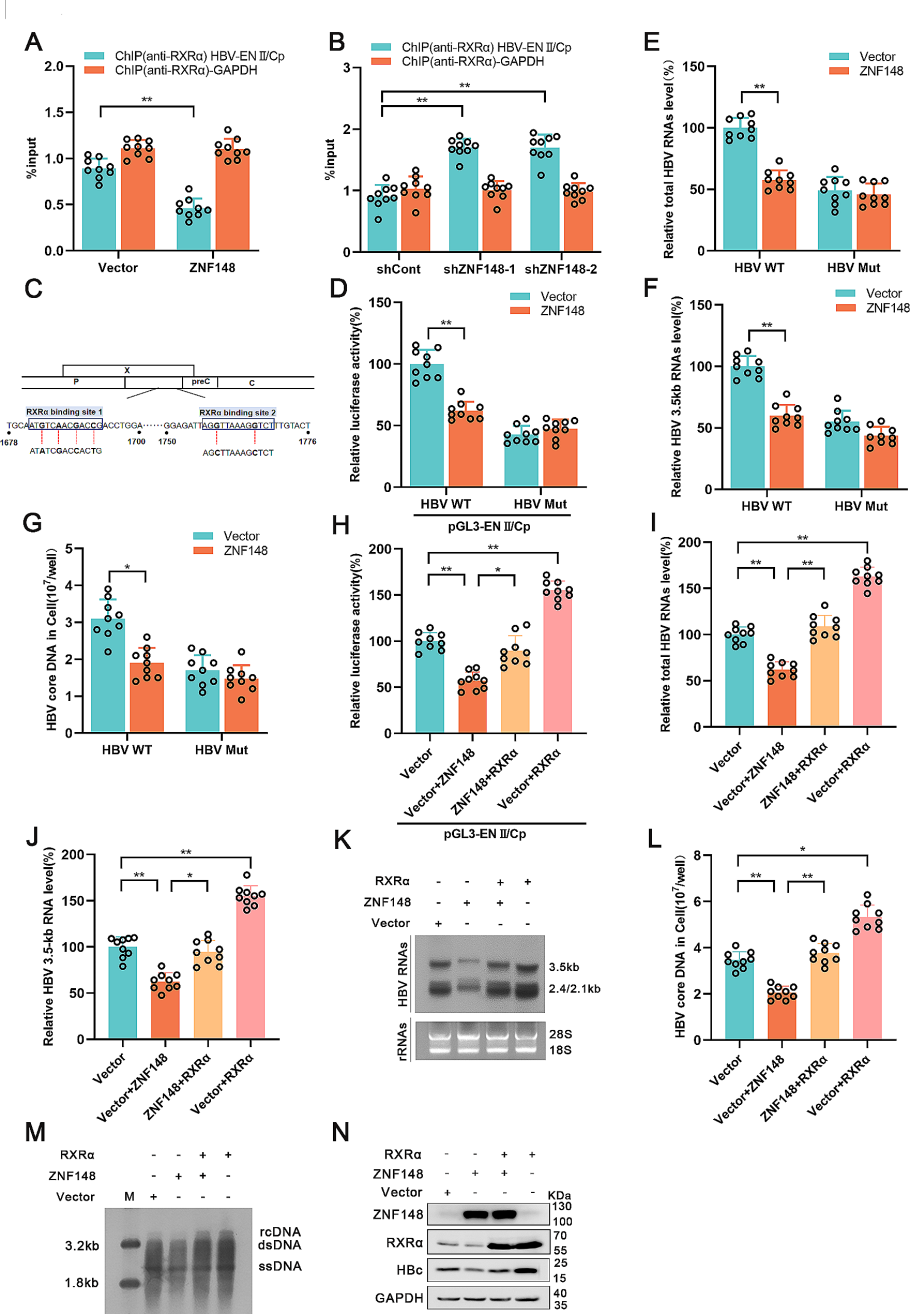
ZNF148 regulates the HBV life cycle by suppressing the binding of RXRα to HBV ENII/Cp. (**A**-**B**) The impact of ZNF148 on the recruitment of RXRα to HBV ENII/Cp was evaluated via a ChIP assay. The promoter of GAPDH served as the control in this analysis. The results are presented as percentages of input. (**C**) The binding sites and mutations are shown in the HBV 1.3 genome. (**D**) The vector/ZNF148 plasmid was cotransfected with the indicated luciferase reporter plasmids into HepG2-NTCP cells. After 36 h, the luciferase activities of pGL3-ENII/Cp and pGL3-ENII/Cp Mut (with mutation of the RXRα binding sites) were measured by **a** dual luciferase assay. (**E**-**G**) HepG2-NTCP cells were transfected with the HBV1.3 genome or the HBV1.3 genomic mut construct (with mutation of the RXRα binding sites); after 24 h, the vector/ZNF148 plasmid was transfected into the cells. The levels of total HBV RNA, 3.5-kb HBV RNA, and HBV core DNA were measured via RT-PCR. (**H**) The ZNF148 or RXRα plasmid was cotransfected with the indicated luciferase reporter plasmids into HepG2-NTCP cells, and luciferase activity was measured using a dual luciferase assay. (**I**-**K**) HepG2-NTCP cells were transfected with the ZNF148 or RXRα plasmid, and HBV RNA levels were measured using real-time PCR and Northern blotting. (**L**-**M**) The HBV core DNA level was measured using real-time PCR and Southern blotting. (**N**) The HBc protein level was measured by Western blotting. *P < 0.05, **P < 0.01

RXRα is a liver-enriched nuclear protein that can interact with other nuclear proteins, such as COUP-TF, FXRα, and PPAR, to bind to genomic transcriptional regulatory elements and enhance the transcriptional activity of target genes [22–24]. RXRα has been confirmed to form heterodimers with PPARα or FXRα and bind to the core promoter of HBV, enhancing the activity of the core promoter [24, 25]. Therefore, we performed a ChIP assay to investigate the impact of ZNF148 on the binding of RXRα to the HBV ENII/Cp region. The binding of RXRα was significantly decreased in ZNF148-overexpressing cells (Fig. 5A). Consistent with this finding, when ZNF148 was silenced, the amount of RXRα bound to the HBV ENII/Cp region was elevated (Fig. 5B). Next, we mutated the RXRα-FXRα-Cp binding site (RXRα binding site 1) and RXRα-PPARα-Cp binding site (RXRα binding site 2) in the HBV1.3 genome (Fig. 5C), which has been previously reported [24, 26]. The impact of ZNF148 on the mutant ENII/Cp promoter was significantly weakened (Fig. 5D). Moreover, the decrease in HBV transcription induced by ZNF148 was abolished when the RXRα binding sites in the HBV core promoter were mutated (Fig. 5E-G). Importantly, the introduction of RXRα attenuated the inhibitory effects of ZNF148 on the activity of ENII/Cp and the HBV RNA, core DNA, and HBc protein levels (Fig. 5H-N). These results indicate that ZNF148 might inhibit HBV replication by suppressing the transcription factor RXRα.

### ZNF148 inhibits HBV infection in vivo

**Fig. 6:**
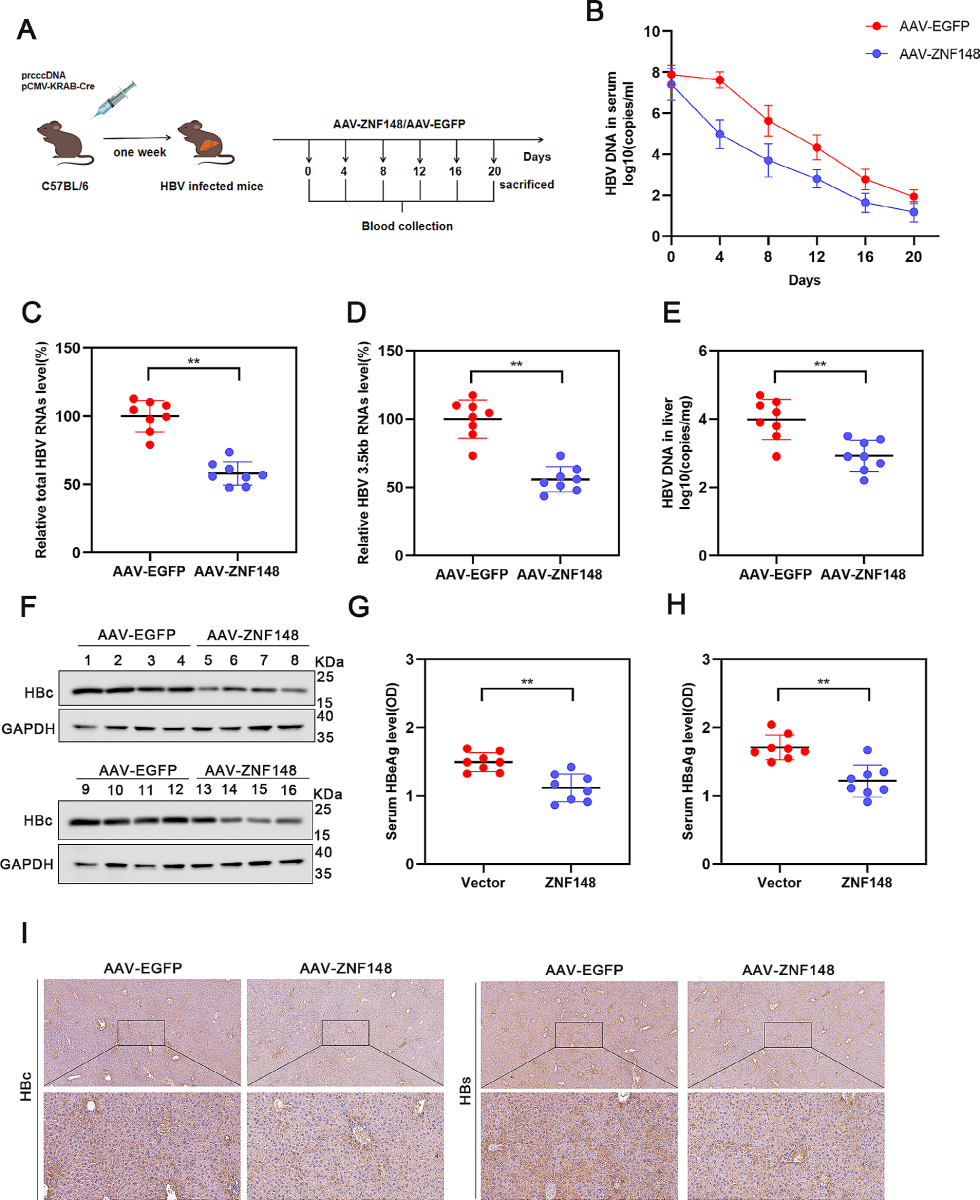
ZNF148 is responsible for regulating the transcription and replication of HBV in vivo. (**A**) A mouse model of HBV infection was established by delivering prcccDNA and Cre plasmids into C57BL/6 mice via high-pressure distal intravenous injection (HDI). Subsequently, successfully infected mice were randomly allocated to the AAV-EGFP group (*n* = 8) or the AAV-ZNF148 group (*n* = 8). (**B**) Real-time PCR was used to measure the level of HBV core DNA in the serum of the mice (**C**-**D**), and the levels of HBV RNAs in the liver were measured using real-time PCR. (**E**) The level of HBV core DNA in the mouse liver was quantified using real-time PCR. (**F**) The expression of the HBc protein in the mouse liver was determined via Western blot analysis. (**G**-**H**) The serum concentrations of HBsAg and HBeAg were measured via ELISA. (**I**) Immunohistochemistry was carried out to examine the expression of HBs and HBc in the mouse liver. *P < 0.05, **P < 0.01

The inhibitory effects of ZNF148 on the HBV life cycle have been demonstrated in cell models, but its functional role in vivo is unclear. To investigate the role of ZNF148 in HBV life cycle in vivo, we generated a mouse model of HBV infection by delivering HBV (r) cccDNA. Furthermore, HBV-infected model mice were treated with 2 × 10^11^ viral genomes of AAV8-ZNF148/AAV-EGFP. Blood samples were collected from the mice every 4 d, and the mice were sacrificed on d 20 (Fig. 6A). Consistent with our expectations, overexpression of ZNF148 resulted in a significant decrease in the serum HBV DNA level (Fig. 6B). After treatment, the mice were sacrificed, and liver tissues were collected to measure the hepatic HBV RNA and DNA levels. ZNF148 overexpression significantly reduced the intrahepatic HBV RNA and DNA levels (Fig. 6C-E). Western blot analysis was performed to demonstrate the efficiency of ZNF148 overexpression via delivery of AAV-ZNF148 in mice (Supplementary Figure S6). ZNF148 overexpression led to a significant reduction in the intrahepatic HBc protein level (Fig. 6F). In addition, compared with control mice, ZNF148-overexpressing mice exhibited significantly lower serum levels of HBeAg and HBsAg (Fig. 6G, H). The intrahepatic HBcAg and HBsAg levels were evaluated by immunohistochemistry, and the results showed that ZNF148 overexpression apparently inhibited the expression of these proteins in liver tissue (Fig. 6I). Collectively, these findings provide strong evidence that ZNF148 effectively suppresses HBV infection in vivo.

## Discussion

ZNF148 is a zinc finger transcription factor, similar to Krüppel (C2H2), that controls the expression of genes by attaching itself to DNA sequences containing GC-rich regions. ZNF148 is essential for controlling genes that are involved in a number of biological processes, such as the development, differentiation, and multiplication of cells [19, 27, 28]. ZNF148 participates in colonic homeostasis by recruiting ataxia-telangiectasia mutated (ATM) to activate the expression of p21 (waf1) [29]. ZNF148 activates erythroid differentiation by regulating the expression of the globin gene [27]. ZNF148 inhibits HCC stemness by suppressing the expression of neurogenic locus notch homolog protein 1 (Notch1) [30]. In this study, we found ZNF148 experession was down-regulated in HBV-infected cells and mice. Subsequently, we identified ZNF148 as an important transcription factor involved in the regulation of HBV transcription. Moreover, the levels of HBV RNAs, HBV core DNA, and the HBc protein, as well as the concentrations of secreted HBeAg and HBsAg in the supernatant, all decreased as a result of our demonstration that ZNF148 inhibited the activity of HBV ENII/Cp. Interestingly, ZNF148 mutant by deleting the zinc finger motif of ZNF148 counteracted the inhibitory role of ZNF48 in HBV replication. These results suggest ZNF148 can inhibit HBV replication depends on its zinc finger motif.

ZFP binding sites have been found in the HBV genome, according to studies [14], and ZNF148 may directly bind to the promoter region of HBV to regulate its transcription. Unfortunately, the results of our ChIP assay revealed that ZNF148 exhibits minimal binding to the HBV genome. This observation suggested that the interaction between ZNF148 and the HBV genome might be indirect or mediated through other factors. It has been established that certain host factors control HBV-related transcription factors, which in turn control the virus’ life cycle. Sirtuin1 regulates transcription and replication by increasing the expression of the transcription factor AP-1, which is an activator of HBV transcription [31]. Spliceosome-associated factor 1 (SART1) suppresses the transcription and replication of HBV by specifically targeting the transcription factor HNF4α [32]. Therefore, we conjectured that ZNF148 may regulate the HBV promoter in conjunction with additional transcription factors. A number of transcription factors that are known to be involved in HBV transcription were examined.

Subsequent examination of these transcription factors demonstrated that ZNF148 bound to the promoter region of RXRα, suppressing the protein’s expression. RXRα is a critical transcriptional activator of HBV ENII/Cp [26], suggesting that RXRα is potentially involved in the regulation of HBV ENII/Cp by ZNF148. It is unknown, yet, how ZNF148 controls the activity of the RXRα promoter. When ZNF148 acts as a repressive transcription factor, it inhibits the effect of activating factors by competitively binding to the same, overlapping, or adjacent DNA elements, resulting in the inhibition of the target gene promoter [33–36]. This occurrence offers valuable information for investigating the mechanism by which ZNF148 controls the activity of the RXRα promoter.

Liver-enriched nuclear protein RXRα can bind to transcriptional regulatory regions in target genes to control their transcription, and it can also form heterodimers with other nuclear factors [22, 23]. The RXRα DNA-binding domain consists of two zinc fingers and three α helices, and the C-terminal α helices are responsible for controlling the DNA-binding function [37]. Previous studies have demonstrated that RXRα interacts with PPARα/FXRα and binds to the core promoter of HBV, leading to an increase in core promoter activity [24]. HepG2-NTCP cells and primary Tupaia hepatocytes have shown increased HBV transcription in response to the RXRα-PPARα heterodimer [38]. The FXRα–RXRα complex can bind to the ENII/Cp sequence and activate viral transcription in Huh7 cells [39]. In the present study, when the binding sites of RXRα-PPARα and FXRα–RXRα in the HBV core promoter were mutated, the inhibitory effect of ZNF148 on the transcription of HBV was abolished. Furthermore, the inhibitory effect of ZNF148 on HBV transcription and replication was lessened by overexpressing RXRα. These findings suggested that RXRα may be crucial for controlling ZNF148 throughout the HBV life cycle.

## Conclusion

In summary, we identified a new transcription factor, ZNF148, which plays a critical role in the HBV life cycle. The inhibitory effect of ZNF148 on HBV replication was demonstrated in vitro and in vivo. Mechanistic studies indicated that ZNF148 suppressed the activity of HBV cccDNA by downregulating RXRα transcription. This study suggested that ZNF148 might be a target for HBV therapy.

### Electronic supplementary material

Below is the link to the electronic supplementary material.

**Supplementary Material 1: Supplementary Table 1.** Primers used in qPCR. Primers used in PCR. Primers used in ChIP-PCR

**Supplementary Material 2: Supplementary Figure 1.** The level of ZNF148 protein was down-regulated after HBV infection. **Supplementary Figure 2.** ZNF148mut ovexpression has little effect on the transcription and replication of HBV. **Supplementary Figure 3.** ZNF148 had the same effect on the activity of ENII/Cp Mut. **Supplementary Figure 4.** ZNF148 exhibits minimal binding to the HBV genome. **Supplementary Figure 5.** Mutation of the binding site of ZNF148 abolished the effect of ZNF148 on the activity of RXR? promoter. **Supplementary Figure 6.** The overexpression effiency of ZNF148 by AAV-ZNF148 was determined by western blot assay

**Supplementary Material 3:** The blot images used in figures

## Data Availability

No datasets were generated or analysed during the current study.

## Abbreviations

ATM: Ataxia-telangiectasia mutated
C/EBP: CCAAT/enhancer-binding protein
cccDNA: Covalently closed circular DNA
ChIP: Chromatin immunoprecipitation
CREB: CAMP response element-binding protein
FXRα: Farnesoid X receptor alpha
HBV: Hepatitis B virus
HNF4α: Hepatocyte nuclear factor 4α
KLF15: Krüppel-like factor 15
Notch1: Neurogenic locus notch homolog protein 1
NTCP: Na^+^/taurocholate cotransporting polypeptide
PHHs: Primary human hepatocytes
RXRα: Retinoid X receptor alpha
SART1: Spliceosome-associated factor 1
STAT1: Signal transducer and activator of transcription family member 1
ZEB2: Zinc-finger E-box binding homeobox 2
ZHX2: Zinc fingers and homeoboxes 2
ZNF148: Zinc finger protein 148
