# Correction: ZNF148 inhibits HBV replication by downregulating RXRα transcription

**DOI:** 10.1186/s12985-024-02420-z

**Published:** 2024-06-25

**Authors:** Xinyan Yao, Kexin Xu, Nana Tao, Shengtao Cheng, Huajian Chen, Dapeng Zhang, Minli Yang, Ming Tan, Haibo Yu, Peng Chen, Zongzhu Zhan, Siyi He, Ranran Li, Chunduo Wang, Daiqing Wu, Jihua Ren

**Affiliations:** 1 The Key Laboratory of Molecular Biology of Infectious Diseases designated by the Chinese Ministry of Education, Chongqing Medical University, Chong Yi Building, 1 YiXueYuan Road, Yuzhong District, Chongqing, 400016 China; 2 Department of Clinical Laboratory, Chongqing Traditional Chinese Medicine Hospital, Chongqing, China; 3 Chongqing Key Laboratory of Sichuan-Chongqing Co-construction for Diagnosis and Treatment of Infectious Diseases Integrated Traditional Chinese and Western Medicine, Chongqing Hospital of Traditional Chinese Medicine, Chongqing, China; 4 Department of Clinical Laboratory, Chongqing Emergency Medical Center, Chongqing University Central Hospital, Chongqing, China

**Correction: Virology Journal (2024) 21:35**

10.1186/s12985-024-02291-4

Following publication of the original article [1], the authors identified an error in Fig. 1H. The result data were mistakenly used when drawing the picture with GraphPad Prism 8.0 software. The authors re-examined the original experiment notes and confirmed that the omission did not affect the conclusions. The correct Fig. 1 is given below:

**Fig. 1:**
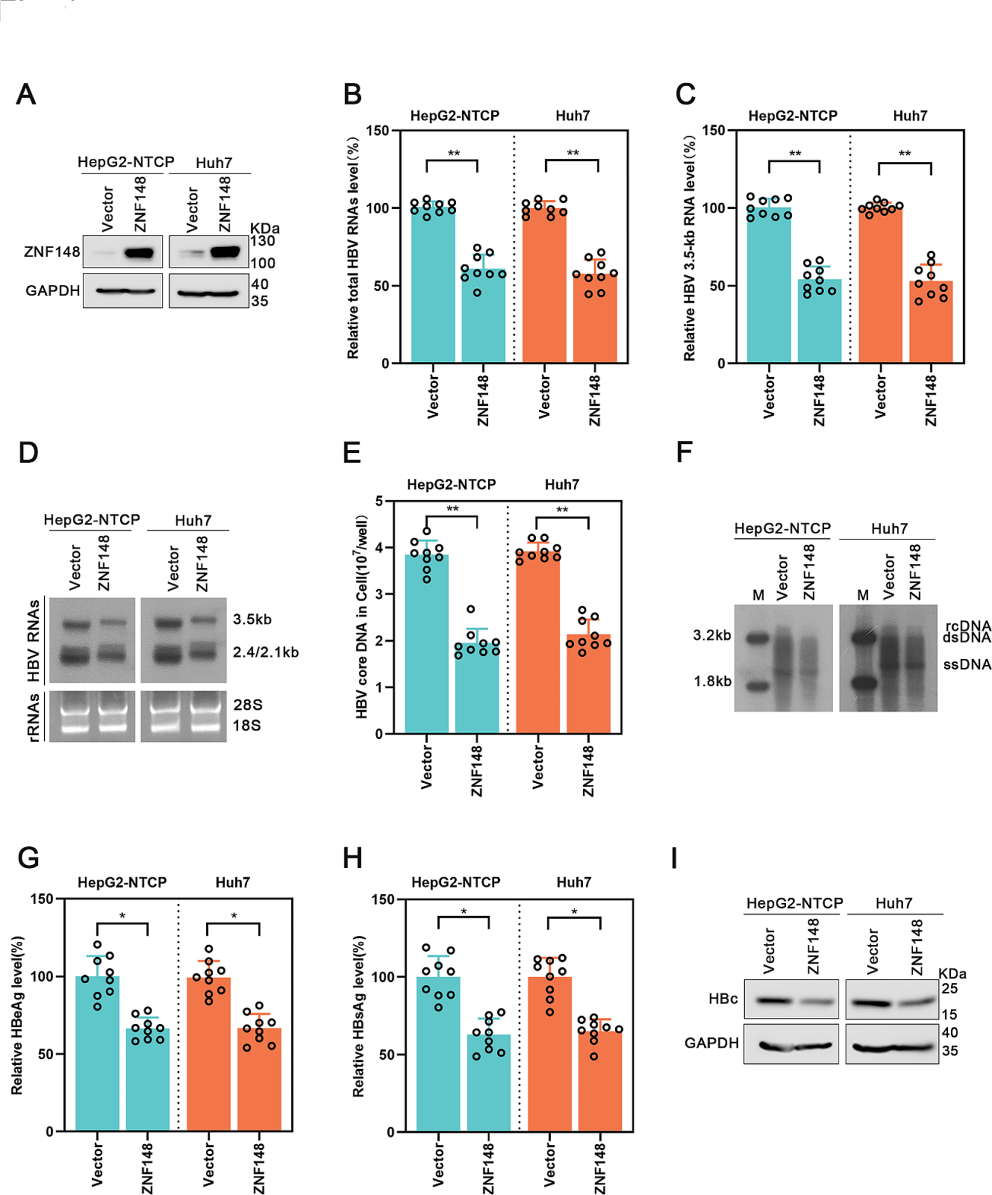
Overexpression of ZNF148 represses HBV transcription and replication. The vector or the ZNF148 plasmid was transfected into HepG2-NTCP cells infected with HBV and into Huh7 cells transfected with prcccDNA/Cre plasmids. The cells were harvested after 5 d. (**A**) The efficiency of ZNF148 overexpression was confirmed by Western blot analysis. (**B**-**D**) Real-time PCR and Northern blot analyses revealed significant reductions in the levels of total HBV RNA and 3.5-kb HBV RNA in cells overexpressing ZNF148. (**E**-**F**) Decreased levels of HBV core DNA in ZNF148-overexpressing cells were shown by real-time PCR and Southern blotting. (**G**-**H**) ZNF148 overexpression reduced the concentrations of HBeAg and HBsAg, as measured by ELISA. (**I**) The results of Western blot analysis confirmed the decreased level of the HBc protein in ZNF148-overexpressing cells. *P < 0.05, **P < 0.01
