# Supplementary material for: ZNF148 inhibits HBV replication by downregulating RXRα transcription
Source: Virol J. 2024 Jan 31;21:35. doi: 10.1186/s12985-024-02291-4 (PMC10832224; doi:10.1186/s12985-024-02291-4)
Supplement: Supplementary file 1 — Supplementary Material 1: Supplementary Table 1. Primers used in qPCR. Primers used in PCR. Primers used in ChIP-PCR [file 12985_2024_2291_MOESM1_ESM.docx]

**Supplementary Table 1**

**Primers used in qPCR**

| HBV core DNA forward | 5’-CCTAGTAGTCAGTTATGTCAAC-3’ |
| --- | --- |
| HBV core DNA reverse | 5’-TCTATAAGCTGGAGGAGTGCGA-3’ |
| total HBV RNAs forward | 5’- ACCGACCTTGAGGCATACTT-3’ |
| total HBV RNAs reverse | 5’- GCCTACAGCCTCCTAGTACA-3’ |
| HBV 3.5-kb RNA forward | 5’- GCCTTAGAGTCTCCTGAGCA-3’ |
| HBV 3.5-kb RNA reverse | 5’- GAGGGAGTTCTTCTTCTAGG-3’ |
| β-actin forward | 5’-CTCTTCCAGCCTTCCTTCCT-3’ |
| β-actin reverse | 5’- AGCACTGTGTTGGCGTACAG-3’ |
| cccDNA forward | 5’- CTCCCCGTCTGTGCCTTCT-3’ |
| cccDNA reverse | 5’-CCCCAAAGCCACCCAAG-3’ |
| cccDNA probe | 5’-TTCATCCTGCTGCTATGCCTGATCTTCTTG-3’ |
| ZNF148 forward | 5’- AGACCTACGACCCACAGGGTGG-3’ |
| ZNF148 reverse | 5’- GGCTTCTCTCCACTGTGAGTT-3’ |
| shZNF148-1 | 5’- GCTACAGGATTCAGTACTTCA-3’ |
| shZNF148-2 | 5’-GGAGATCCTTGCTGCAGATGA-3’ |
| shCont | 5′-GCAACAAGATGAAGAGCACCAA-3′ |
| PPARα forward | 5’-TGGAGCATTGAACATCGAAT-3’ |
| PPARα reverse | 5’-GGTCGCACTTGTCATACACC-3’ |
| Sp1 forward | 5’-CTGAAGCTGGGTAGCCTATTG-3’ |
| Sp1 reverse | 5’-CTACTGCTGCGACCTTTCTT-3’. |
| FXRα forward | 5’-TGTGAGGGGTGTAAAGGTTTCT-3’ |
| FXRα reverse | 5’-GCCAACATTCCCATCTCTTTGC-3’ |
| ZHX2 forward | 5’-AAGGTCCCTGTCCCACTAAA -3’ |
| ZHX2 reverse | 5’-TGACTGGATGGTGCCGTTGA-3’ |
| p53 forward | 5’-AACAACACCAGCTCCTCTCC-3’ |
| p53 reverse | 5’-CTCATTCAGCTCTCGGAACA-3’ |
| HNF3α forward | 5’-CAGCAAACAAAACCACACAAACC-3’ |
| HNF3α reverse | 5’-ACACTTGTGGATCATTAAACTTCGC-3’ |
| HNF3β forward | 5’-GTTGTTGTTGTTCTCCTCCATTGC-3’ |
| HNF3β reverse | 5’-AACTACATGGTTTTACACCGAGTCAC-3’ |
| C/EBP α forward | 5’-CGAGCCAGGACTAGGAGATT-3’ |
| C/EBP α reverse | 5’-CCTCATCTTAGACGCACCAA-3’ |
| C/EBP β forward | 5’-CTGGAGACGCAGCACAAG-3’ |
| C/EBP β reverse | 5’-ACAGCTGCTCCACCTTCTTC-3’ |
| KLF15 forward | 5’-CCAAGTTCAGCCGCCACC-3’ |
| KLF15 reverse | 5’-GGCATTTTGGCGACGAGAAG-3’ |
| HNF4α forward | 5’-GCCTACCTCAAAGCCATCAT-3’ |
| HNF4α reverse | 5’-CGGTCGTTGATGTAGTCCTC-3’ |
| TR4 forward | 5’-TTGTGAAGGTTGCAAAGGTT-3’ |
| TR4 reverse | 5’-TTCATGCCCATCTCTAAGCA-3’ |
| COUP-TF forward | 5’-ACTGCTACCTGTCCGGCTAC-3’ |
| COUP-TF reverse | 5’-TCGATGCCCATAATGTTGTT-3’ |
| CREB forward | 5’-CAGTTATTCAGTCTCCACAAG-3’ |
| CREB reverse | 5’-CTTCTCTTCTTCAATCCTTGG-3’ |
| MIBP1 forward | 5’-CAGGAATAACCCGGTGCGT-3’ |
| MIBP1 reverse | 5’-AATTCAAAGGCTCCACCGAG-3’ |
| ZEB2 forward | 5’-CCTCTGTAGATGGTCCAGTGA-3’ |
| ZEB2 reverse | 5’-GTTCCAGGTGGCAGGTCATT-3’ |
| RXRα forward | 5’-GGACATGCAGATGGACAAGA-3’ |
| RXRα reverse | 5’-CCCTTGGAGTCAGGGTTAAA-3’ |
| RFX1 forward | 5’-GCAACGCGAGTCAACAACAA-3’ |
| RFX1 reverse | 5’-GCATGGCACCTTCAGAGACA-3’ |

**Primers used in PCR**

| ZNF148 Mut forward | 5’-CCTAAATCGAAAATCATGACAAAAAAC-3’ |
| --- | --- |
| ZNF148 Mut reverse | 5’-GATTTTCGATTTAGGGGTTTTCAAAC-3’ |
| HBV EnⅡ/Cp Mut forward | 5’-GGGAAGAAATTAGGTTAAAGGTCTTTGTACTAG-3’ |
| HBV EnⅡ/Cp Mut reverse | 5’-CTAATTTCTTCCCCCAACTCCTCCCAG-3’ |
| RXRα promoter Mut forward | 5’-AGTGAAGAGAGGGCTGCCTTGGGCTCTGG-3’ |
| RXRα promoter Mut reverse | 5’-AGCCCTCTCTTCACTGACCCCTGGGC-3’ |

**Primers used in ChIP-PCR**

| HBV EnⅡ/Cp forward | 5’-CCACCAAATATTGCCCAAGG-3’ |
| --- | --- |
| HBV EnⅡ/Cp reverse | 5’-AGCCTCCTAGTACAAAGACC-3’ |
| GAPDH forward | 5’-TACTAGCGGTTTTACGGGCG-3’ |
| GAPDH reverse | 5’-TCGAA AGGAGGAGCAGAGAGCGA-3’ |
| RXRα forward | 5’-CTCTCCCTGCACTGAGCATA-3’ |
| RXRα reverse | 5’-GAACAACCACTCCCTCCTCA-3’ |
