# Supplementary material for: ZNF148 inhibits HBV replication by downregulating RXRα transcription
Source: Virol J. 2024 Jan 31;21:35. doi: 10.1186/s12985-024-02291-4 (PMC10832224; doi:10.1186/s12985-024-02291-4)
Supplement: Supplementary file 2 — Supplementary Material 2: Supplementary Figure 1. The level of ZNF148 protein was down-regulated after HBV infection. Supplementary Figure 2. ZNF148mut ovexpression has little effect on the transcription and replication of HBV. Supplementary Figure 3. ZNF148 had the same effect on the activity of ENII/Cp Mut. Supplementary Figure 4. ZNF148 exhibits minimal binding to the HBV genome. Supplementary Figure 5. Mutation of the binding site of ZNF148 abolished the effect of ZNF148 on the activity of RXR? promoter. Supplementary Figure 6. The overexpression effiency of ZNF148 by AAV-ZNF148 was determined by western blot assay [file 12985_2024_2291_MOESM2_ESM.docx]

**Figure S1**

**
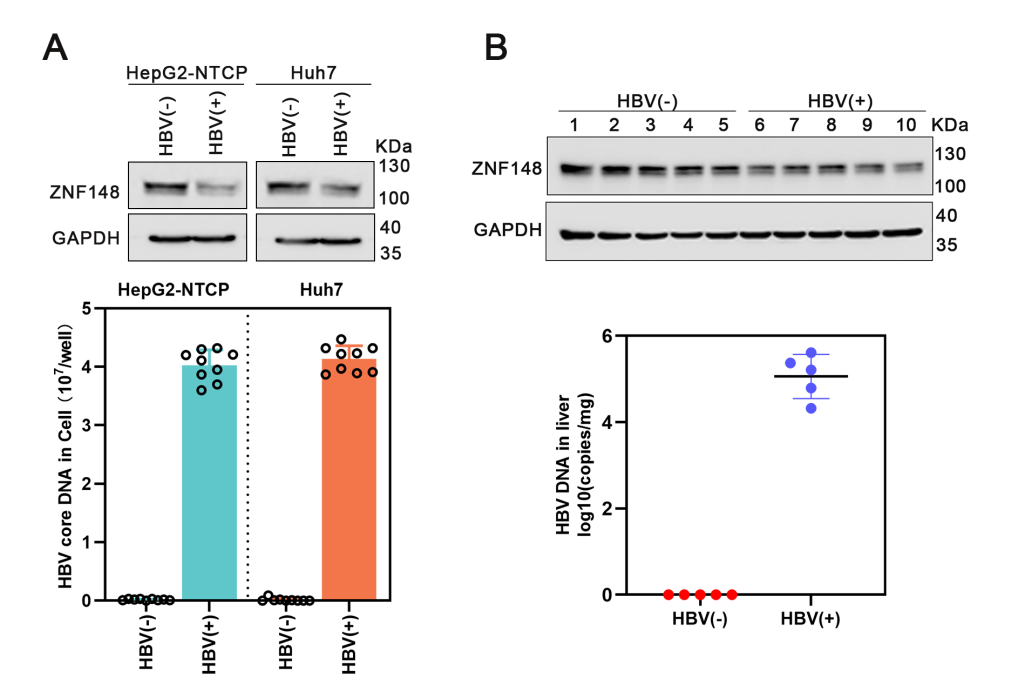
**

**Supplementary Figure 1. The level of ZNF148 protein was down-regulated after HBV infection.** (A) HepG2-NTCP cells were transfected with HBV, Huh7 cells were transfected with prcccDNA/Cre plasmids, after 5 d, the cells were harvest. Western blot assay showed that the level of ZNF148 protein was down-regulated in HBV-infected HepG2-NTCP cells and Huh7 cells expressing prcccDNA. The level of HBV DNA in both cell lines was showed by Real-time PCR.(B) 10 C57BL/6 mice were randomly allocated to the HBV(-) group (n=5) or the HBV(+) group (n=5), then, the mice of HBV(+) group were delivered into prcccDNA and Cre plasmids via high-pressure distal intravenous injection. After two weeks, the mice were sacrificed, the western blot assay showed that the level of ZNF148 protein in mice liver was down-regulated after HBV infection. The level of HBV DNA in mice liver was showed by Real-time PCR.

**Figure S2**

**
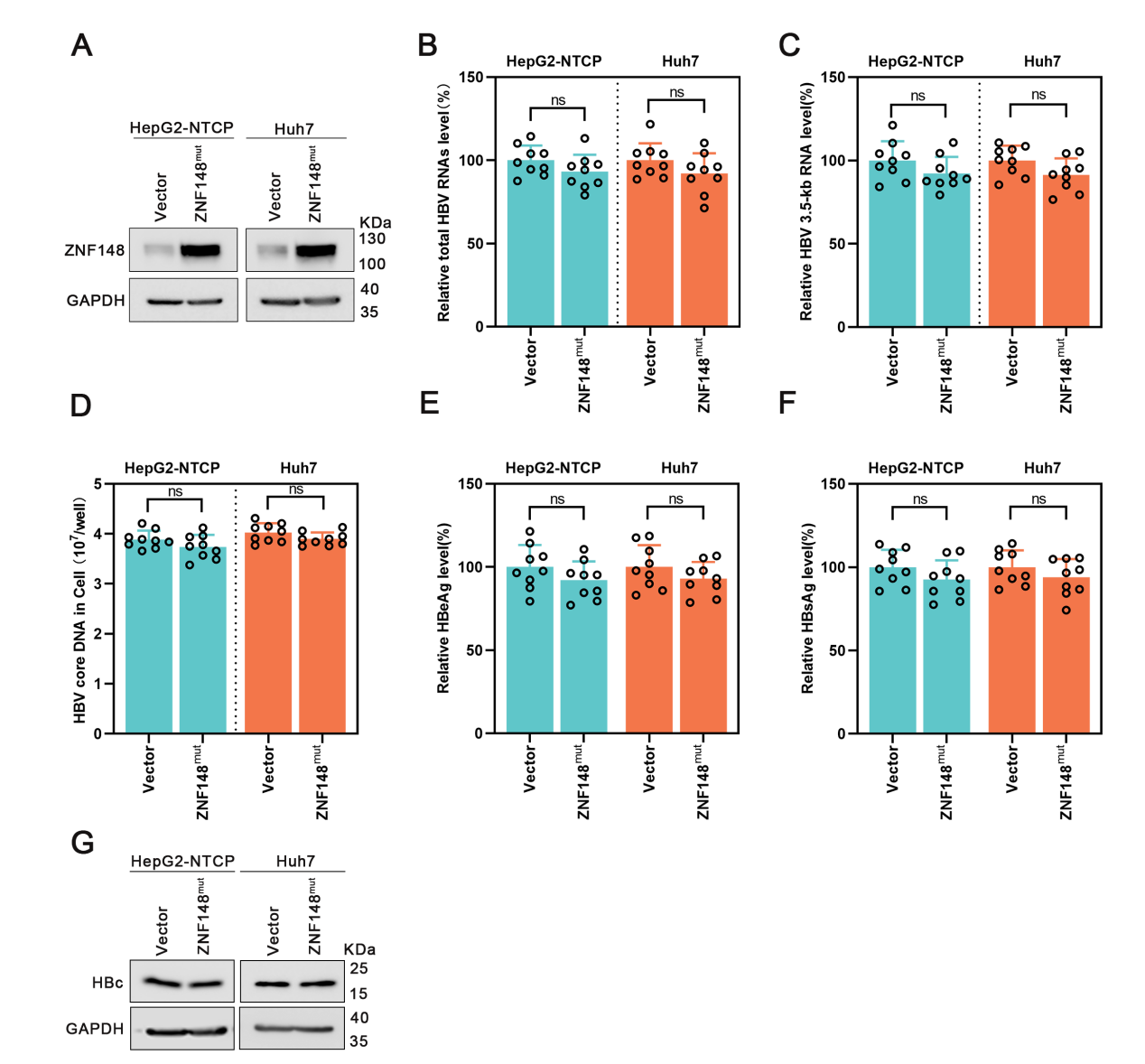
**

**Supplementary Figure 2. ZNF148^mut^ ovexpression has little effect on the transcription and replication of HBV.** The vector or the ZNF148 plasmid was transfected into HepG2-NTCP cells infected with HBV and into Huh7 cells transfected with prcccDNA/Cre plasmids. The cells were harvested after 5 d. (A) The efficiency of ZNF148^mut^ overexpression was confirmed by Western blot analysis. (B, C) Real-time PCR analysis revealed little effect on the levels of total HBV RNA and 3.5-kb HBV RNA in cells overexpressing ZNF148^mut^. (D) Real-time PCR analysis revealed little effect on the levels of HBV core DNA in ZNF148^mut^-overexpressing cells. (E, F) ZNF148^mut^ overexpression has little effect on the concentrations of HBsAg and HBeAg, as measured by ELISA. (G) The results of Western blot analysis confirmed that ZNF148^mut^ overexpression has little effect on level of the HBc protein.

**Figure S3**

**
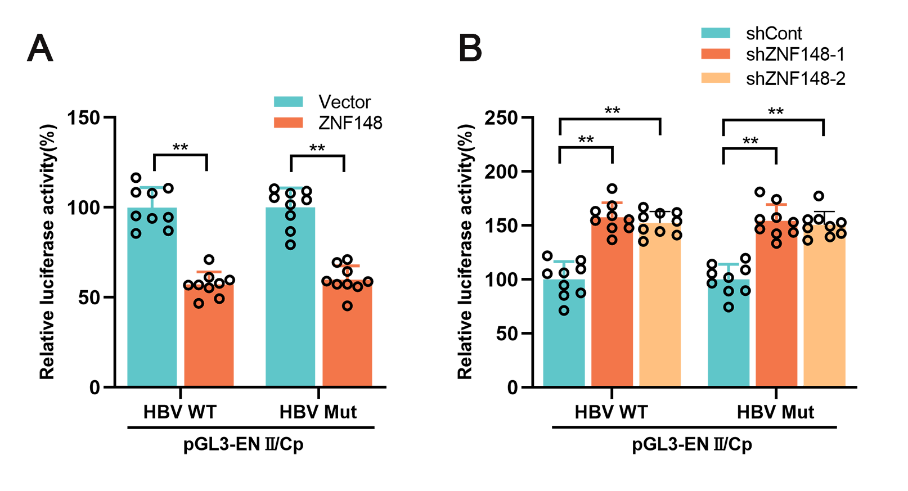
**

**Supplementary Figure 3. ZNF148 had the same effect on the activity of ENⅡ/Cp Mut.** HepG2-NTCP cells were transfected with HBV pGL3-ENⅡ/Cp or HBV pGL3-ENⅡ/Cp Mut (ZNF148 binding site was mutated). Then vector/ZNF148 plasmids or shCont/shZNF148 were transfected in the cells to overexpress or knockdown ZNF148. (A) Overexpression of ZNF148 had the same inhibitory effect on the activity of ENⅡ/Cp WT with the activity of ENⅡ/Cp Mut. (B) Silencing of ZNF148 had the same enhanced effect on the activity of ENⅡ/Cp WT with the activity of ENⅡ/Cp Mut.

**Figure S4**


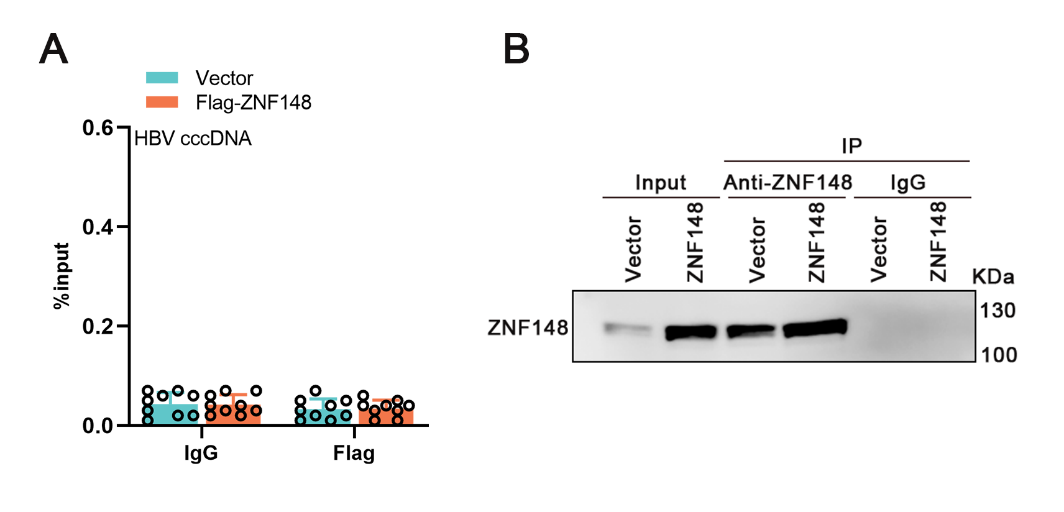


**Supplementary Figure 4. ZNF148 exhibits minimal binding to the HBV genome.** HepG2-NTCP cells were transfected with vector/ZNF148 plasmids, (A) ChIP assay was performed to determine whether ZNF148 binded to the HBV genome. (B) Western blot assay was conducted to confirm that ZNF148 was immunoprecipitated in the ChIP experiments.

**Figure S5**


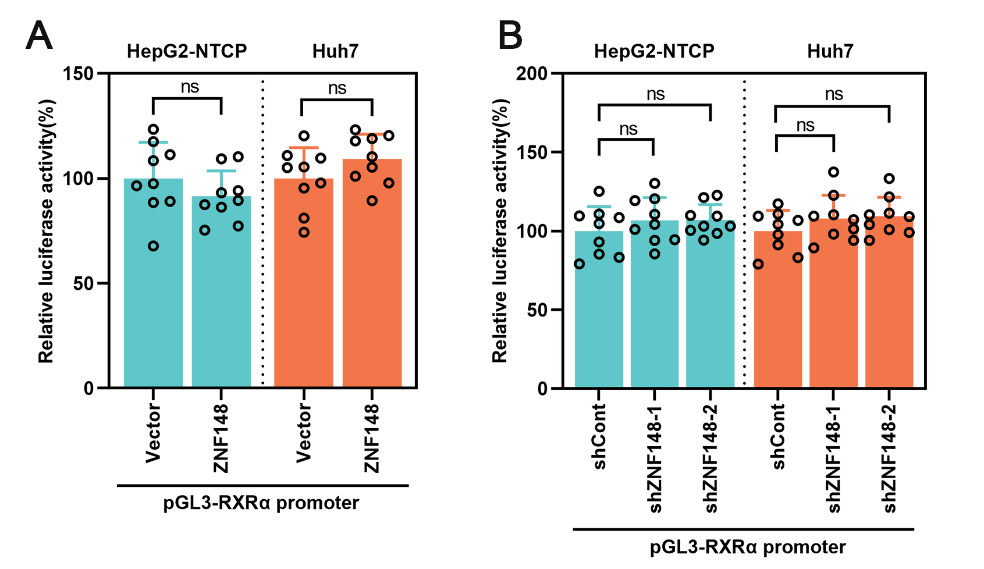


**Supplementary Figure 5. Mutation of the binding site of ZNF148 abolished the effect of ZNF148 on the activity of RXRα promoter**. (A) HepG2-NTCP and Huh7 cells was transfected with pGL3-RXRα promoter Mut (ZNF148 binding site was mutated), then vector/ZNF148 plasmids were transfected into the cells, after 36 h, the luciferase activity was determined by dual-luciferase reporter assay. (B) HepG2-NTCP and Huh7 cells was transfected with pGL3-RXRα promoter Mut, the shCont/shZNF148 were transfected into the cells, after 36 h, the luciferase activity was determined by dual-luciferase reporter assay.

**Figure S6**


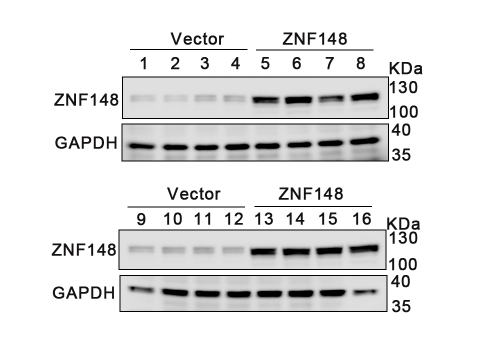
’

**Supplementary Figure 6. The overexpression effiency of ZNF148 by AAV-ZNF148 was determined by western blot assay**. HBV-infected model mice were treated with 2×10^11^ viral genome AAV8-ZNF148/AAV-EGFP. After 20 d, the mice were sacrificed. The protein level of ZNF148 in mice liver were determined by western blot assay. GAPDH was used as loading control.
