# Supplementary figures and images for: ZNF148 inhibits HBV replication by downregulating RXRα transcription
Source: Virol J. 2024 Jan 31;21:35. doi: 10.1186/s12985-024-02291-4 (PMC10832224; doi:10.1186/s12985-024-02291-4)

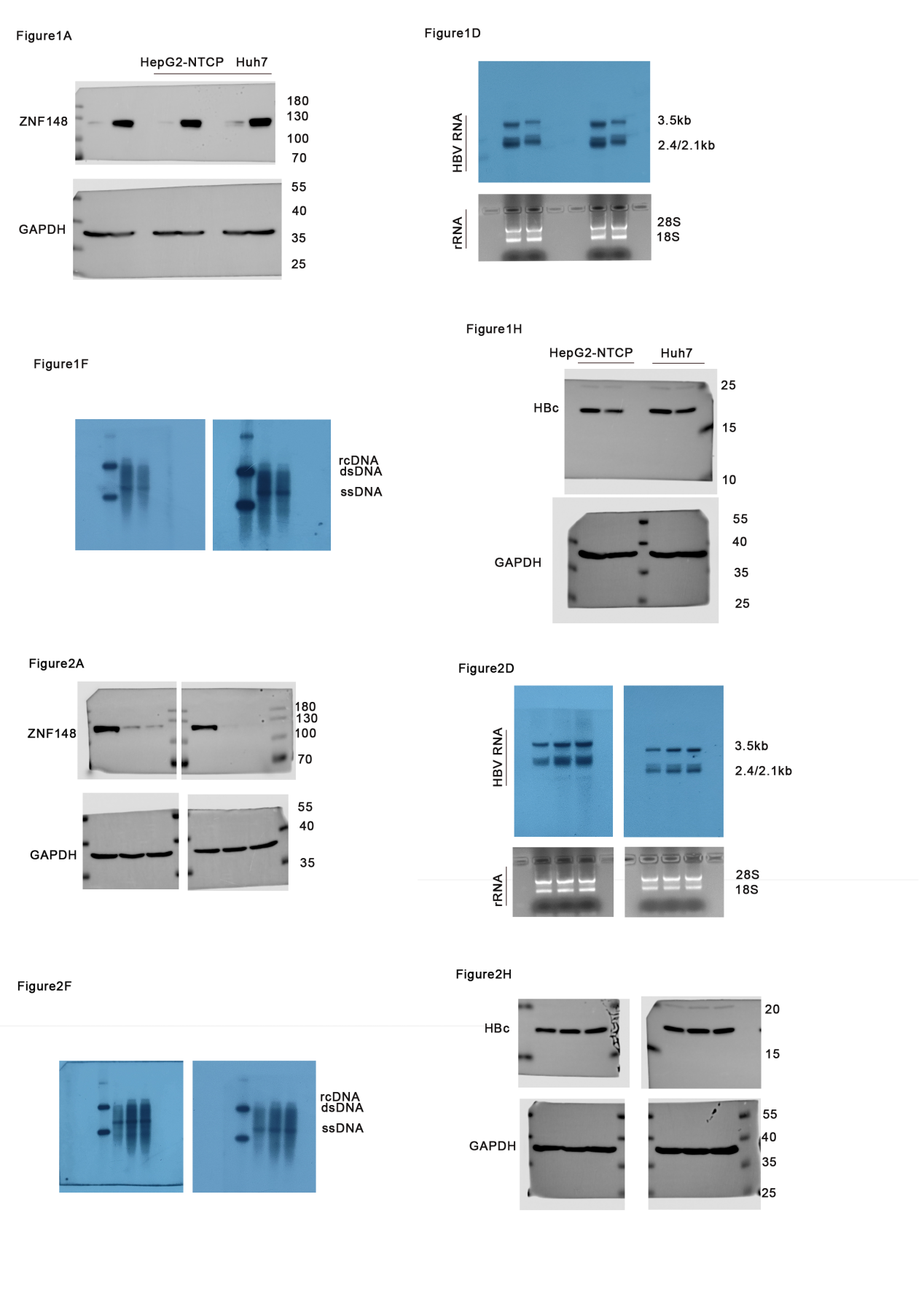

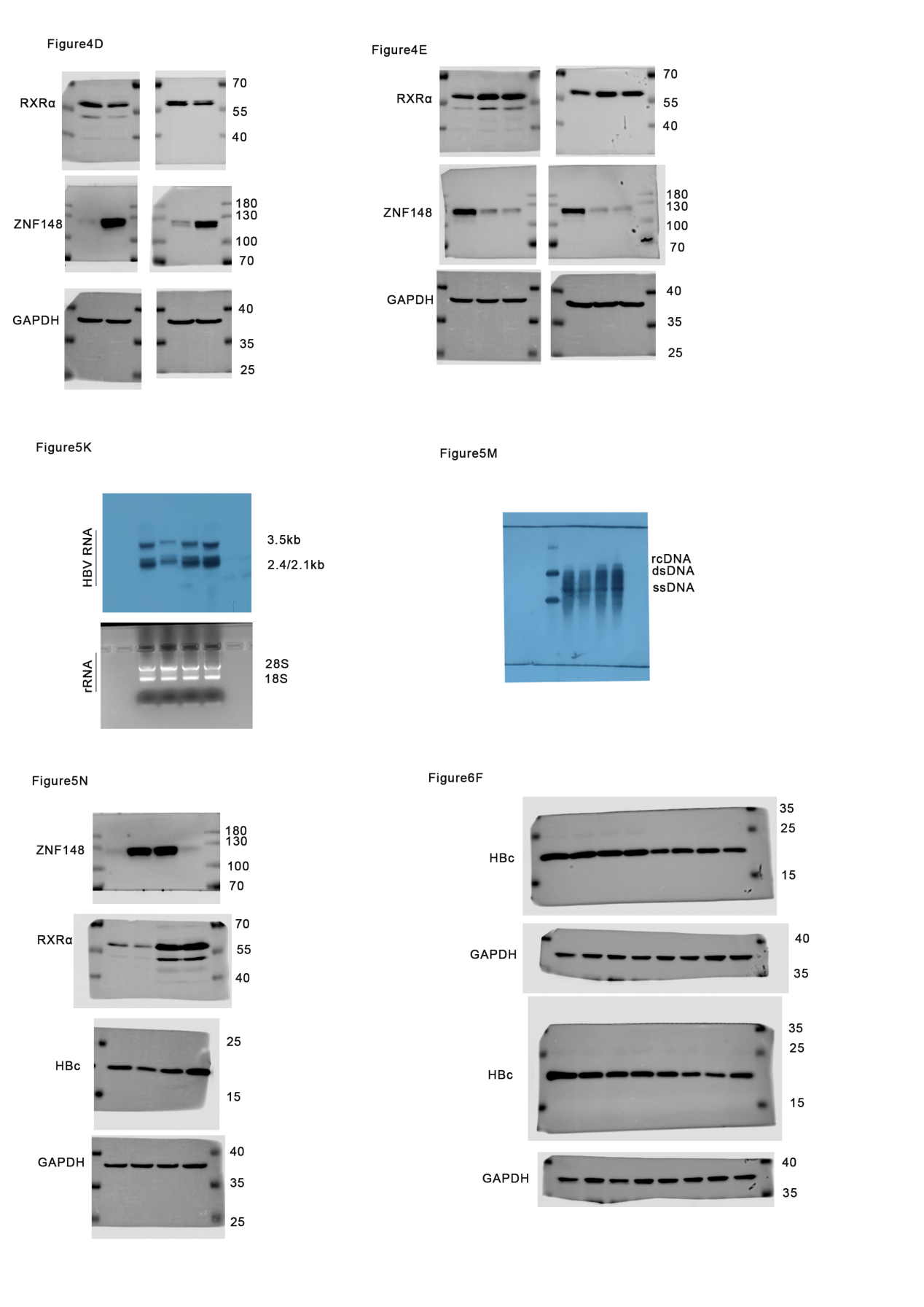


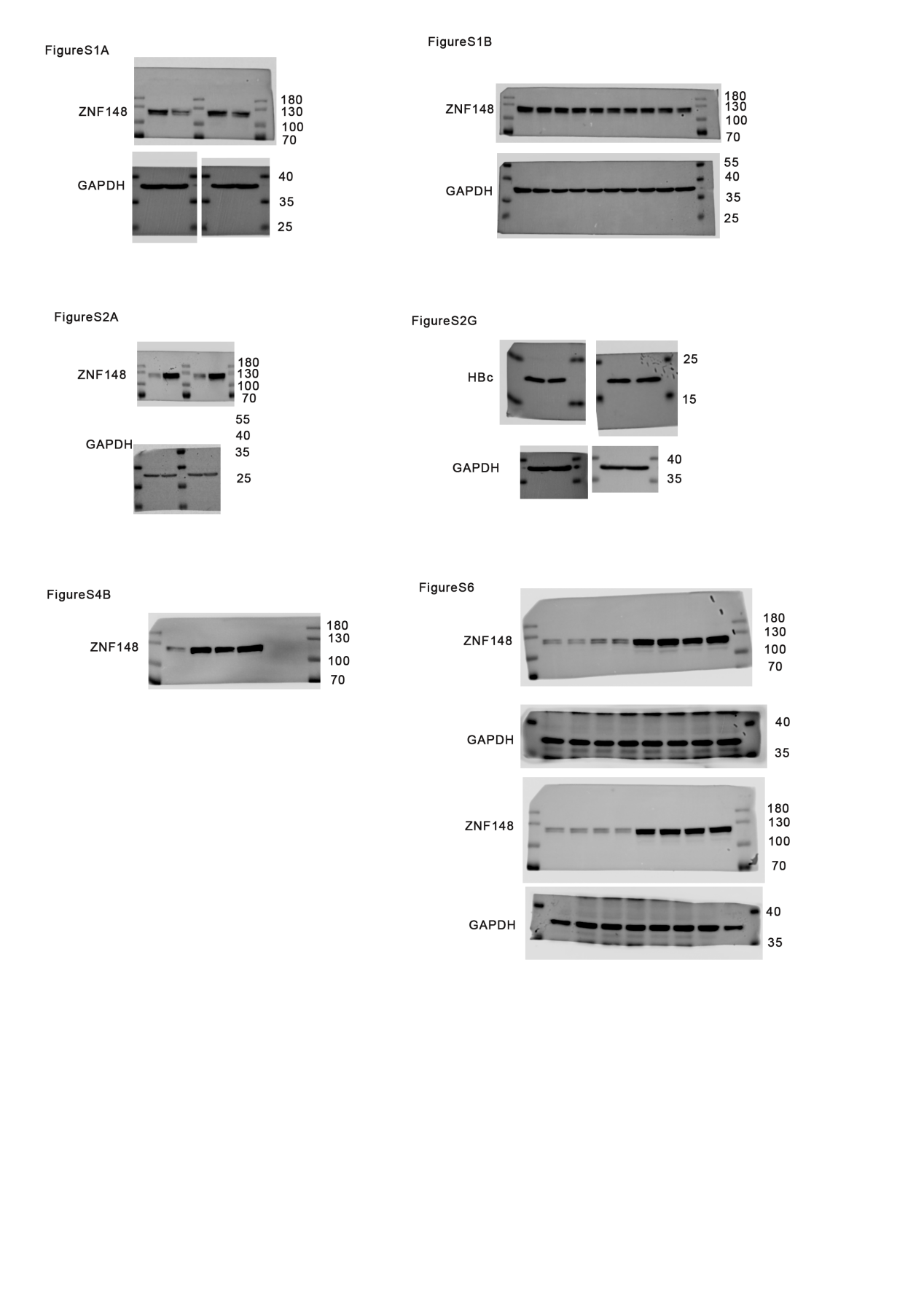

Supplement: Supplementary file 3 — Supplementary Material 3: The blot images used in figures [file 12985_2024_2291_MOESM3_ESM.docx]
